# Modeling the interactive development of sports services and the silver economy in aging societies

**DOI:** 10.3389/fpubh.2026.1777277

**Published:** 2026-03-31

**Authors:** Quanchen Zhou, Lide Su, ZhiXiang Wen

**Affiliations:** College of Physical Education, Hunan Normal University, Changsha, China

**Keywords:** aging societies, dynamic panel analysis, physical activity, silver economy, sports services

## Abstract

**Background:**

Rapid population aging is reshaping preventive health priorities and the silver economy, yet the dynamic linkage between sports services and aging-related economic activity remains underexplored.

**Methods:**

Using multi-regional panel data, we constructed validated composite indices for sports services and the silver economy and estimated bidirectional dynamic relationships with system GMM, testing mediation via older adults’ physical activity and moderation by aging intensity and health awareness.

**Results:**

Sports services development positively predicted subsequent silver economy growth, and silver economy expansion also fed back to strengthen sports services. Physical activity participation partially mediated the effect, while stronger impacts appeared in more aged and higher health-awareness contexts, with evidence of persistence and non-linearity.

**Conclusion:**

Sports services operate as a preventive health infrastructure that supports active aging and contributes to silver economy development through dynamic, behavior-linked feedback mechanisms.

## 1 Introduction

Population aging has emerged as a new demographic trait in both the developed and developing regions reconfiguring health systems (1), labor markets (2), and consumption patterns (3). With the rising population over 65, societies face growing challenges related to functional decline and the high prevalence of chronic diseases (4), and the surging healthcare spending (5). A factor found to be one of the critical risks, on top of the challenges (6), has been identified as physical inactivity enhancing the challenges in the aging populations due to a decrease in mobility, loss of independence and also the quality of life (7). The need to provide structured, accessible, and age-appropriate sports services and physical activity has therefore increased significantly in the aging societies within this context (8). The idea of silver economy has therefore changed significantly in this regard. The silver economy has always been traditionally focused on medical treatment and long-term care, which is now marked by a transition to preventive health, active aging, and lifestyle-based consumption (9). It is a sign of a more generalized theoretical redirection of the healthcare expenditure towards the health maintenance and functional capacity investment. Involvement in moderate exercising and use of sports services (10) have become the core ingredients of this prevention paradigm and physical activity is considered not only a health behavior but also an economic factor contributor in the societies in old age. The sports services form an important subsystem of the larger infrastructure of the public health and aging support (11).

A substantial body of research in sports science and preventive medicine shows that regular participation in well-designed physical activity programs supports the maintenance of muscle strength (12), balance, and cardiovascular functions in older adults (13), as well as decreases the risk of non-communicable diseases, including type 2 diabetes, osteoporosis (14), and cardiovascular diseases (15). In addition to being found to be physiologically beneficial, sports and exercise-based services are linked to enhanced mental health, social involvement and life satisfaction in older populations (16). The use of sports services to encourage older adults to exercise is not only limited to individual motivation, but also to the nature and availability of service delivery.

Basic access and equity has its fundament underpinnings with the presence of public sports services, especially to older adults with socioeconomic disadvantages (17). The sports services of commercial benefit by enhancing diversification and specialization and providing customized programs in response to the heterogeneous preferences and functional capabilities. The community based services promote social interaction and long term participation (18), whereas the digital and technology based sports services increases access by minimizing both spatial and time based limitations (19). The interaction of these various ways of provision influences physical activity behaviors among older people and affects their participation levels and long term adherence. Though there is a common understanding that sports services are one of the aspects of healthy aging, their position in larger dynamics of silver economy is under-theorized and under-empirically studied (20). In terms of the interactive development, sports services and silver economy can be formulated to view as mutually reinforcing systems (21).

On the one hand, the growth of sports service delivery provokes the increased demand to the goods and services of active aging connected to the increase of physical capacity, health awareness, and engagement in leisure and wellness activities (22). Conversely, the silver economy is growing, which creates market demand and financial resources that promote the diversification, professionalization, and upgrading of sports services technologically. This two-way interaction is facilitated by health capital that is gained with time. When older people maintain a constant involvement in sports services, it leads to enhanced functional health (23), which in turn facilitates their further economic involvement and consumption. Nevertheless, existing empirical evidence has addressed mostly these associations separately and either looked at the health implications of exercise or looked at the economic aspects of the aging related sectors (24). A literature that explicitly indicates the development of the services and the silver economy in the line of dynamism and interaction is still scarce, especially through the prism of sports science that assumes a combination of health behavior, service delivery, and economic performance in an analytical system. Based on this context, this paper will review the relationship and co-relation between sports services and the silver economy in aging communities.

This paper examines three interdependent questions within the institutional context of China: how regional development of sports services relates to subsequent development of the silver economy, whether expansion of the silver economy is associated with later improvements in the scale and quality of sports services, and the extent to which older adults’ physical activity participation functions as a behavioral link between the two systems. Addressing these questions requires an approach that can capture reciprocal dynamics and temporal ordering rather than treating sports services and aging-related markets as independent domains. Accordingly, we adopt a system-oriented empirical strategy using multi-dimensional indicators of sports services and silver economy development and exploit regional panel variation to examine lagged and interactive relationships. Drawing on health capital theory and life-course health development, we conceptualize sports services as an investable preventive-health input that supports the accumulation of functional health stock through sustained physical activity behavior, providing a theoretically grounded basis for the proposed dynamic linkages.

## 2 Methods

### 2.1 Study design and data sources

The study assumed a longitudinal panel design based on multi-regional observations and the years of observation were continuous and consecutive. An analytical sample consisted of 31 regions (2011 to 2022), which gave an equal balance of 372 region-year observations. Because all observations are drawn from regions within China, the estimates capture within-country variation under a shared national institutional framework, which constrains cross-national generalization. The regional unit of analysis was suitable in terms of capturing spatial heterogeneity in sports service provision, demographic aging and economic development as well as providing adequate statistical strength to do dynamical modelling.

The data on supplying the services of sports services were gathered through the regional sports statistical yearbooks and administrative records, such as records about sports facilities, service institutions, and certified personnel. Information on older adults’ physical activity participation was drawn from China’s population health and lifestyle survey program, which reports the percentage of residents aged ≥60 years who engage in regular physical activity. To ensure comparability, we used survey waves implemented under the same core questionnaire and fieldwork protocol across all 31 regions and harmonized the indicator to a consistent definition of “regular physical activity” over the study period; where wording or sampling adjustments occurred, values were aligned using the survey’s recommended cross-wave coding and documented harmonization rules. The indicators regarding silver economy, such as consumption expenditure, health related industry output and leisure and fitness related spending which are age specific, were derived using the official economic and industrial statistics. The yearbooks of the local health statistics were used in the acquisition of the yearbook health expenditure and healthcare resource indicators.

Physical activity participation (PA). PA participation was measured as the percentage of adults aged ≥60 who met the primary survey’s criterion for “regular exercise.” In the primary data sources, regular exercise was defined using a standardized frequency-duration threshold (e.g., engaging in moderate-intensity or higher physical activity on ≥3 days per week and ≥30 min per session, or an equivalent weekly volume consistent with public-health recommendations). To maximize comparability across the 31 regions and the study period, we harmonized all survey waves to a common definition by (i) mapping wave-specific response options to a consistent frequency and duration threshold, and (ii) applying the same recoding rules across regions. When minor changes in wording or response categories occurred, we followed the survey’s recommended cross-wave harmonization procedures and documented the recoding scheme in the data processing protocol.

Table 1 records the descriptive results to the key variables employed in the empirical study. The both sports service development and silver economy indicators show a significant regional and temporal variation, which in turn confirms the appropriateness of using the dataset to investigate both interactive and heterogeneous effects.

**Table 1:** Descriptive statistics of main variables (*N* = 372).

Variable	Mean	Std. Dev.	Min.	Max.
Sports services development index	0.487	0.163	0.152	0.892
Silver economy development index	0.521	0.171	0.184	0.903
Physical activity participation rate (≥60 years, %)	42.36	11.48	18.2	71.5
Sports facilities per 10,000 residents	15.84	6.27	4.9	34.6
Certified sports instructors per 10,000 residents	2.91	1.34	0.62	7.85
Per capita GDP (10,000 CNY)	7.26	3.91	2.14	19.84
Population aged ≥60 years (%)	18.94	4.72	9.8	32.1
Health expenditure per capita (CNY)	3,284	1,462	1,120	8,940
Urbanization rate (%)	59.73	14.68	32.1	89.4

### 2.2 Operationalization of variables

To ensure analytical clarity and comparability across regions, all core variables were operationalized using standardized and widely adopted indicators. Composite indices were constructed to reflect the multidimensional nature of both sports services development and the silver economy.

The sports services development index captured the availability, accessibility, and professional capacity of sports services. Facility density measured the number of public and commercial sports facilities per 10,000 residents. Service accessibility reflected the coverage of community-level sports service outlets and the average service radius. Professional staffing was measured by the number of certified sports instructors per 10,000 residents. Program diversity reflected the range of age-appropriate physical activity programs offered to older adults. Indicators were standardized and aggregated to form a composite index.

The silver economy development index captured economic activities associated with aging populations, with particular attention to distinguishing wellness-oriented demand from disease-treatment expenditure. It included (i) per capita consumption expenditure of older adults, (ii) value added in older adult-oriented industries, and (iii) spending on leisure, fitness, and sports-related services. For the health-related component, we prioritized indicators that better reflect preventive and health-maintenance demand (e.g., wellness services, rehabilitation/functional training, preventive health products and services) rather than aggregating total medical spending. Where only aggregate health expenditure was available, we treated it as a control variable and/or decomposed it—when data permitted—into a preventive/wellness-related share versus treatment-related expenditure using the statistical classification of expenditures/industries. All components were normalized and combined to form the composite index, reducing the likelihood that increases driven by disease burden or inefficiency are misinterpreted as silver-economy “development.”

Several control variables were included to account for confounding demographic and structural factors. Population aging was measured as the proportion of residents aged 60 years and above. Economic development was captured by per capita GDP. Healthcare resource availability was measured using per capita health expenditure and the number of hospital beds per 10,000 residents. Urbanization level was measured as the proportion of the population living in urban areas. Table 2 provides detailed definitions and measurement methods for all variables.

**Table 2:** Variable definitions and measurement.

Variable	Definition	Measurement	Data source
Sports services development index	Overall level of sports service provision	Composite index (standardized)	Sports statistical yearbooks
Facility density	Availability of sports facilities	Facilities per 10,000 residents	Sports administration records
Service accessibility	Ease of access to sports services	Community coverage (%)	Regional statistics
Professional staffing	Sports service capacity	Certified instructors per 10,000 residents	Sports administration records
Program diversity	Variety of sports programs for older adults	Number of program categories	Sports service reports
Silver economy development index	Development level of aging-related economy	Composite index (standardized)	Economic statistical yearbooks
Older adults’ consumption	Consumption level of older population	Per capita expenditure (≥60 years)	Household surveys
Health-related industry output	Scale of aging-related industries	Value added (CNY)	Industrial statistics
Physical activity participation	Exercise engagement of older adults	% aged ≥60 meeting the survey-defined regular exercise criterion (≥3 times/week and ≥30 min/session or ≥ 90 min/week moderate-equivalent)	Health surveys
Population aging	Demographic structure	% aged ≥60 years	Census data
Economic development	Regional economic level	Per capita GDP	Statistical yearbooks
Healthcare resources	Medical service availability	Beds per 10,000 residents	Health yearbooks
Urbanization	Level of urban development	Urban population (%)	Statistical yearbooks

Based on the above analysis, this study puts forward the following hypotheses:

*H1 (Health capital investment path)*: Sports services development predicts subsequent growth of the silver economy, partly through increased physical activity participation (investment behavior).

*H2 (Behavioral mechanism)*: Sports services development positively predicts older adults’ physical activity participation due to improved opportunity structures (ecological model).

*H3 (Feedback/derived demand)*: Silver economy expansion predicts subsequent upgrades in sports services provision via derived demand and fiscal/market capacity.

*H4 (Life-course/accumulation)*: The impact of sports services on the silver economy exhibits lagged and cumulative effects consistent with health capital accumulation.

*H5 (Context conditioning)*: Aging intensity and health awareness moderate the marginal effect of sports services on silver economy outcomes.

### 2.3 Research framework

The analytical framework guiding this study conceptualizes sports services as a preventive health infrastructure that shapes older adults’ behavioral responses and, through these responses, contributes to the development of the silver economy. In this framework, sports services development is treated as a multi-dimensional system encompassing facility provision, accessibility, staffing capacity, and program diversity. These service conditions operate as environmental enablers that lower participation barriers and increase opportunities for sustained engagement in physical activity. Physical activity participation is positioned as an observable behavioral pathway through which investments in sports services may translate into health-related capability maintenance—captured conceptually as health capital—thereby supporting continued consumption, market participation, and demand in aging-related sectors.

**Figure 1:**
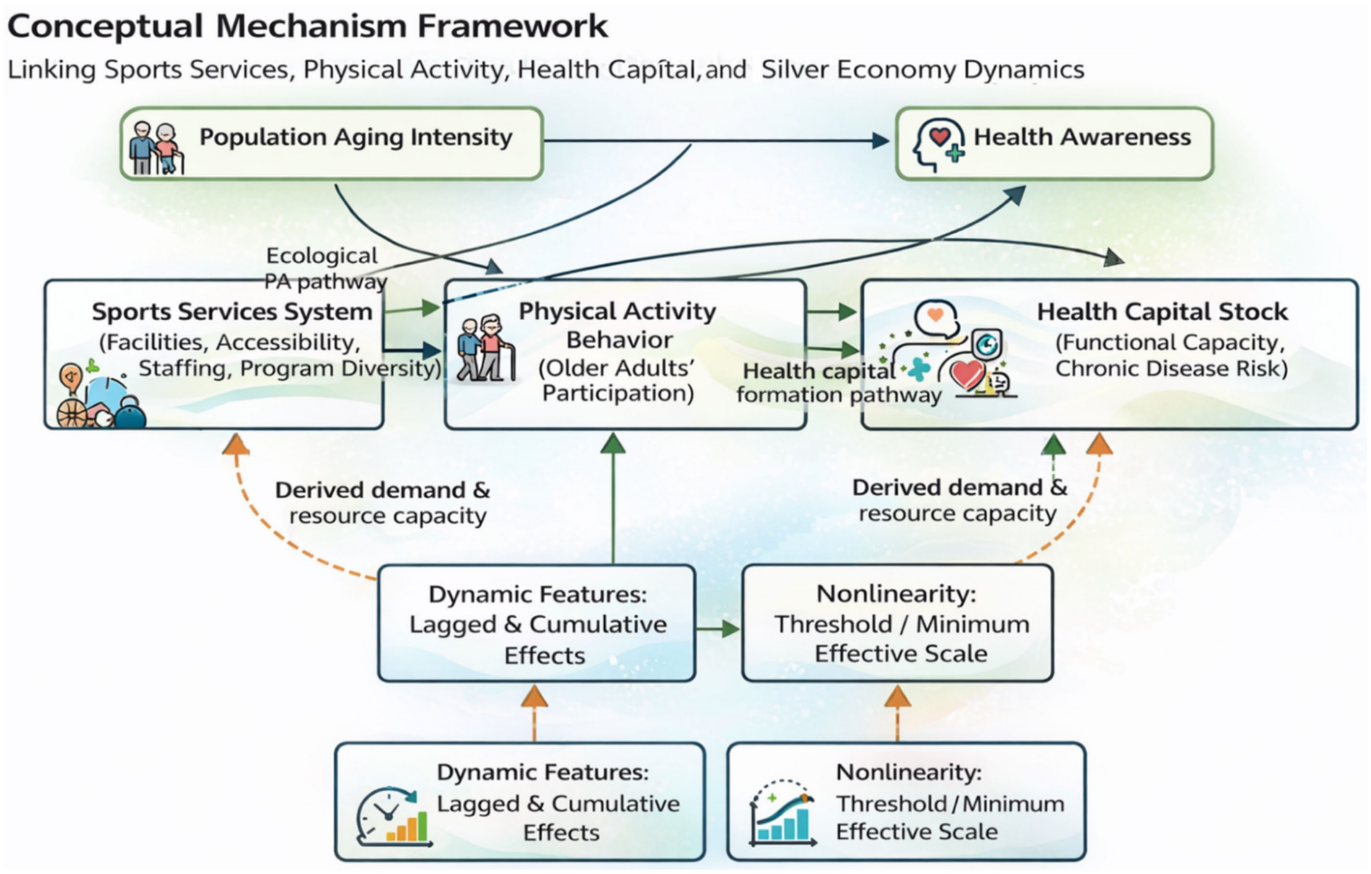
Conceptual mechanism framework linking sports services, physical activity, health capital, and silver economy dynamics.

At the same time, the framework allows for reciprocal dynamics. Expansion of the silver economy is expected to increase resource capacity and derived demand for sports services, encouraging service upgrading and diversification over time. Contextual factors such as population aging intensity and health awareness are incorporated as moderators that may strengthen or weaken the marginal effects of sports services on behavior and downstream outcomes, while dynamic features (lagged and cumulative effects) and nonlinearity (threshold or minimum effective scale) capture realistic adjustment processes in service systems and markets. This integrated set of hypothesized pathways and moderating conditions is summarized in the conceptual framework presented as Figure 1.

### 2.4 Modeling strategy: dynamic interaction modeling of sports services and the silver economy

The empirical strategy was designed to identify bidirectional and time-dependent linkages between sports services development and the silver economy in aging societies. Because both systems can plausibly influence each other and exhibit persistence over time, the analysis adopted a dynamic panel framework with reciprocal equations. This approach allows (i) estimation of lagged (path-dependent) effects, (ii) explicit modeling of feedback loops, and (iii) mitigation of bias from unobserved time-invariant regional characteristics through fixed effects and appropriate instrumentation.

Let i=1,…,N index regions and t=1,…,T index years. The key constructs are: a sports services development index ${\mathrm{SS}}_{\mathrm{it}}$ , a silver economy development index ${\mathrm{SE}}_{\mathrm{it}}$ , and a vector of control variables $X_{\mathrm{it}}$ (e.g., population aging share, per capita GDP, healthcare resources, urbanization). The baseline reciprocal dynamic specification is:


$${\mathrm{SS}}_{\mathrm{it}}=\alpha_{0}+\rho \;{\mathrm{SS}}_{i,t-1}+\beta \;{\mathrm{SE}}_{i,t-1}+\gamma^{\prime }X_{\mathrm{it}}+\mu_{i}+\lambda_{t}+\varepsilon_{\mathrm{it}},$$ (1)

$${\mathrm{SE}}_{\mathrm{it}}=\delta_{0}+\phi \;{\mathrm{SE}}_{i,t-1}+\theta \;{\mathrm{SS}}_{i,t-1}+\kappa^{\prime }X_{\mathrm{it}}+\eta_{i}+\tau_{t}+\nu_{\mathrm{it}}.$$ (2)

In Equation 1, ρ captures persistence in sports services development, while β quantifies whether prior silver economy expansion predicts subsequent improvement in sports services. In Equation 2, ϕ reflects persistence in the silver economy index, and θ estimates whether prior sports services development predicts later silver economy growth. Region fixed effects $\mu_{i},\eta_{i}$ address time-invariant heterogeneity (e.g., geography, long-standing institutional differences), and year fixed effects $\lambda_{t},\tau_{t}$ capture common shocks and national trends (e.g., macroeconomic cycles, national policy shifts).

#### 2.4.1 Interaction effects and lag structure

To test whether the effect of sports services depends on the intensity of aging or other structural conditions, interaction terms were introduced. Using population aging share ( ${\mathrm{Age}}_{\mathrm{it}}$ , defined as the proportion aged ≥60 ) as a theoretically grounded moderator, Equation 2 was extended as:


$$\begin{array}{l} {\mathrm{SE}}_{\mathrm{it}}=\delta_{0}+\phi \;{\mathrm{SE}}_{i,t-1}+\theta \;{\mathrm{SS}}_{i,t-1}+\omega ({\mathrm{SS}}_{i,t-1}\times {\mathrm{Age}}_{\mathrm{it}})\\ +\kappa^{\prime }X_{\mathrm{it}}+\eta_{i}+\tau_{t}+\nu_{\mathrm{it}}. \end{array}$$ (3)

Here, ω captures whether sports service development produces stronger (or weaker) silver economy gains in more aged regions, which is consistent with demand-side amplification mechanisms and differential health-capital returns across demographic contexts.

Lagged effects were defined deliberately. One-year lags (t−1) were used as the primary structure to reflect the realistic time needed for (i) changes in service supply to influence behavior and spending patterns, and (ii) market growth to translate into service investment, staffing, and program diversification. In sensitivity analyses, longer lags (e.g., t−2 ) were tested to capture slower-moving adjustments in infrastructure and industry structure.

#### 2.4.2 Optional mechanism model: physical activity as a mediating pathway

To evaluate whether older adults’ physical activity participation ( ${\mathrm{PA}}_{\mathrm{it}}$ , % of individuals aged ≥60 who exercise regularly) acts as a mechanism linking sports services to the silver economy, a dynamic mediation structure was specified:


$${\mathrm{PA}}_{\mathrm{it}}=a_{0}+a_{1}\;{\mathrm{PA}}_{i,t-1}+a_{2}\;{\mathrm{SS}}_{i,t-1}+a_{3}^{\prime }X_{\mathrm{it}}+\xi_{i}+\psi_{t}+u_{\mathrm{it}},$$ (4)

$${\mathrm{SE}}_{\mathrm{it}}=b_{0}+b_{1}\;{\mathrm{SE}}_{i,t-1}+b_{2}\;{\mathrm{SS}}_{i,t-1}+b_{3}\;{\mathrm{PA}}_{i,t-1}+b_{4}^{\prime }X_{\mathrm{it}}+\eta_{i}+\tau_{t}+\nu_{\mathrm{it}}.$$ (5)

The indirect (mediated) effect of sports services on the silver economy is quantified as $a_{2}\times b_{3}$ . In this setting, lagging PA in Equation 5 supports a temporally ordered mechanism consistent with behavioral change preceding economic outcomes.

Physical activity participation is used as an observable behavioral mediator; however, it does not capture the full spectrum of health behaviors and social participation that may respond to sports services (e.g., preventive care engagement, health-oriented consumption preferences, or community social connectedness). Therefore, the mediated effect estimated here should be interpreted as the portion of the sports-services effect that operates through measured physical activity, not the total behavioral mechanism.

### 2.5 Statistical analysis: estimation, diagnostics, robustness, and identification

#### 2.5.1 Estimation approach

The presence of lagged dependent variables and reciprocal dynamics raises concerns about Nickell bias under fixed effects estimation, as well as potential endogeneity from reverse causality and omitted time-varying shocks. Therefore, Equations 1–3 were estimated using dynamic panel generalized method of moments (GMM), with region fixed effects eliminated through transformation and lagged variables used as internal instruments.

The baseline estimator was the Arellano–Bover/Blundell–Bond system GMM, which combines equations in first differences and in levels to improve instrument strength when variables are persistent. Under standard assumptions of no serial correlation in the idiosyncratic error term beyond first order, valid moment conditions include:

For the Equation 6:


$$E[{\mathrm{SS}}_{i,t-s}\;\Delta \varepsilon_{\mathrm{it}}]=0,E[{\mathrm{SE}}_{i,t-s}\;\Delta \nu_{\mathrm{it}}]=0,s\geq 2,$$ (6)

and for the level equation (system component):


$$E[\Delta {\mathrm{SS}}_{i,t-1}\;(\mu_{i}+\varepsilon_{\mathrm{it}})]=0,E[\Delta {\mathrm{SE}}_{i,t-1}\;(\eta_{i}+\nu_{\mathrm{it}})]=0.$$ (7)

In Equation 7, ${\mathrm{SS}}_{i,t-2}$ and deeper lags were used as instruments for $\Delta {\mathrm{SS}}_{i,t-1}$ , and ${\mathrm{SE}}_{i,t-2}$ and deeper lags were used as instruments for $\Delta {\mathrm{SE}}_{i,t-1}$ . Control variables were treated according to their plausibility of endogeneity: strictly exogenous controls (e.g., national time dummies) were entered directly, while potentially endogenous controls (e.g., health expenditure) were instrumented using appropriate lag structures.

#### 2.5.2 Model diagnostics and fit

For GMM estimation, model adequacy and identification were assessed using established diagnostic tests:

• Serial correlation tests on differenced residuals (Arellano–Bond):
 o AR(1) is expected due to differencing;
 o Absence of AR(2) provides support for instrument validity in dynamic specifications.
• Over-identification tests (Hansen J test; and Sargan as a sensitivity check under homoskedasticity assumptions) to evaluate the joint validity of instruments:

$$H_{0}:\text{instruments}\;\mathrm{are}\;\text{exogenous}.$$ (8)

- Instrument proliferation control to avoid overfitting endogenous variables and weakening the Hansen test (Equation 8). Instruments were restricted by limiting lag depth and collapsing instrument matrices when appropriate.

For the mediation component (Equations 4, 5), the indirect effect $a_{2}\times b_{3}$ was evaluated using bootstrap-based confidence intervals adapted to panel settings, with resampling at the regional unit to preserve within-region temporal dependence.

#### 2.5.3 Multicollinearity, endogeneity, and heterogeneity handling

Multicollinearity was examined prior to estimation using variance inflation factors (VIF) for the time-varying regressors included in $X_{\mathrm{it}}$ . Where high collinearity was detected among conceptually overlapping controls, variables were reparameterized (e.g., using per capita GDP rather than multiple income proxies) to maintain interpretability without inflating standard errors. Endogeneity was addressed at three levels. The dynamic structure itself reduces simultaneity by relating outcomes to lagged predictors. More importantly, system GMM explicitly instruments the lagged dependent variables and potentially endogenous regressors using deeper lags, mitigating reverse causality between sports services and the silver economy. As an additional robustness check, alternative identification choices were tested by (i) treating a broader set of controls as endogenous, and (ii) using alternative lag ranges for instruments. Heterogeneity was assessed through both interaction modeling and subgroup analyses. Interaction terms (Equation 3) tested whether the marginal effect of sports services varied with population aging intensity, consistent with demand-side and health-capital mechanisms in aging societies. Complementary stratified analyses were performed by splitting regions into high-aging versus low-aging groups based on the median aging share, re-estimating the main equations to examine whether effect sizes were stable across demographic contexts.

#### 2.5.4 Robustness and sensitivity analyses

Robustness was evaluated using multiple complementary strategies:

Alternative index construction: recalculating ${\mathrm{SS}}_{\mathrm{it}}$ and ${\mathrm{SE}}_{\mathrm{it}}$ using equal-weight aggregation and principal component analysis (PCA) to verify that results were not driven by a specific weighting scheme.
Alternative lag structures: replacing one-year lags with two-year lags for key predictors to test sensitivity to slower adjustment processes.
Alternative estimators: comparing system GMM estimates with fixed-effects models and bias-corrected least squares dummy variable approaches (where applicable) to assess consistency in direction and magnitude.
Outlier influence checks: re-estimating models after winsorizing extreme observations of economic variables (e.g., per capita GDP) to confirm that results were not dominated by a small number of high-income regions.

Across specifications, statistical significance was evaluated using two-sided tests with conventional thresholds, and standard errors were made robust to heteroskedasticity and within-region serial correlation. This analytical protocol was chosen to ensure that reported effects reflect stable associations consistent with the hypothesized interactive development mechanism rather than artifacts of model specification or data irregularities.

Because the data are observational, coefficients are interpreted as conditional associations consistent with the theorized causal structure; lagging key predictors, controlling for fixed effects, and applying system GMM are used to mitigate simultaneity and endogeneity, but they do not fully eliminate the possibility of time-varying unobserved confounding.

## 3 Results and discussion

### 3.1 Distributional characteristics and correlation structure of key variables

**Figure 2:**
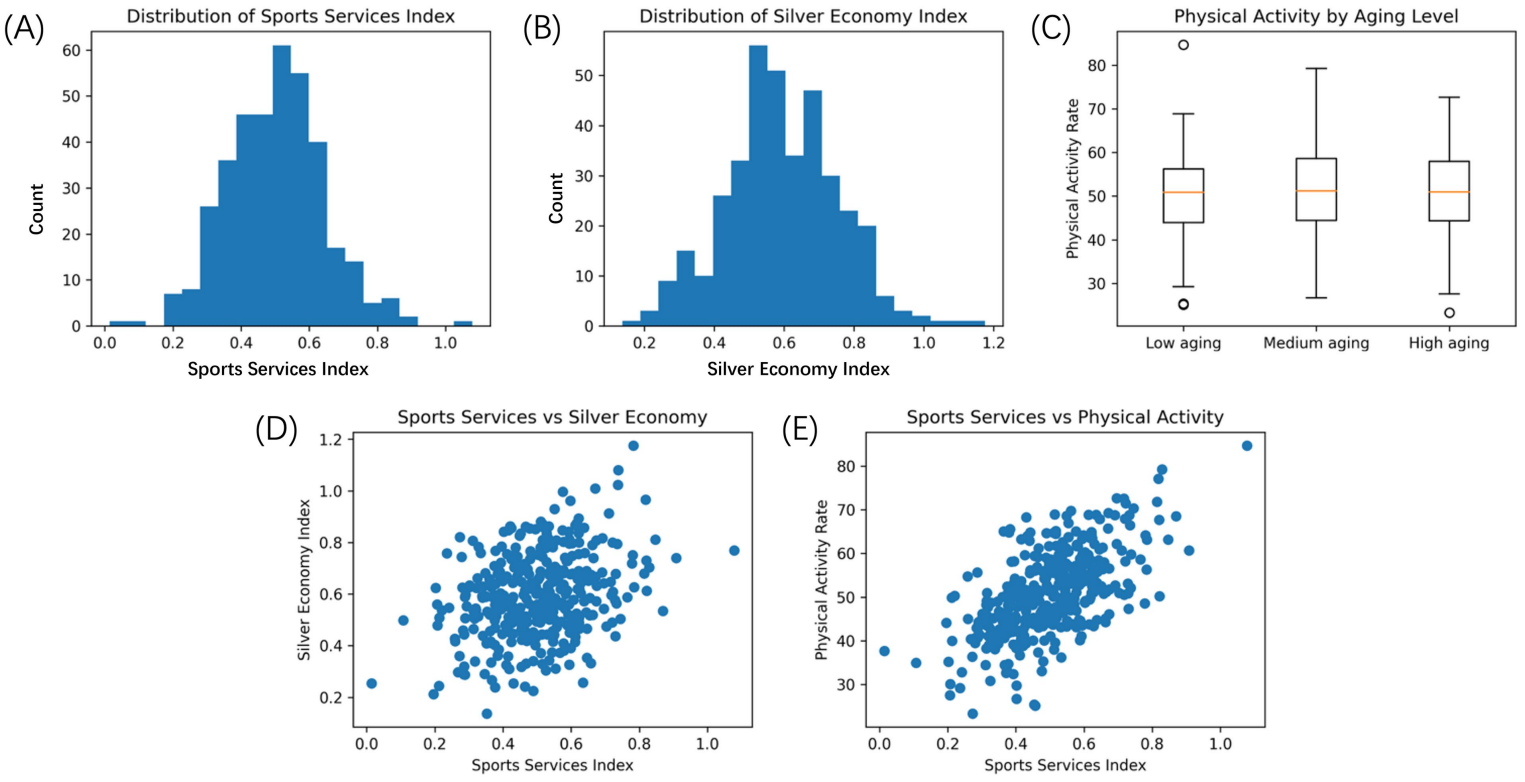
Descriptive statistics and correlation structure of key variables. **(A)** Shows the distribution of the sports services development index. **(B)** The distribution of the silver economy development index. **(C)** Boxplots of older adults’ physical activity participation rates across regions with low, medium, and high aging intensity. **(D)** The bivariate relationship between sports services development and silver economy development. **(E)** The association between sports services development and physical activity participation among older adults.

The descriptive analysis shows that there is a significant cross-regional and temporal difference in both the development of sports services and the silver economy. As shown in Figure 2A the development index of sports services has a moderately homogeneous distribution with the majority of the values concentrated towards the mid range although there is an apparent right tail. This trend suggests that most regions have developed sports services to a certain basic level; however, a smaller number of regions have experienced relatively high levels of development representing uneven distribution of the infrastructure investment and the service capacity of regions. The same tendency of distribution is represented by the index of development of the silver economy (Figure 2B). Though the distribution is also concentrated in and around the mean, the upper tail indicates that there are areas which have undergone a rapid growth in aging associated economic activities especially in the health, leisure and fitness based sectors. The similarity in the distributional forms of the two indices can serve as a preliminary indication that areas that have a stronger sports service systems have a greater likelihood to be at a higher level of development in a silver economy, which needs to be formally modeled.

These are further demographic aging trends that are identified through grouped descriptive statistics. The distribution of participation rates of older adults in the physical activity given the regions in terms of the intensity of aging is reported in Figure 2C. Areas with greater older populations exhibit statistically better median physical activity engagement with smaller ranges of interquartiles. This tendency gives reasons to believe that more older adult areas are more likely to have a higher investment in age-related sport services or that older populations are the ones that have more stable participation frameworks due to the long-term shift in the demographic situation.

Figures 2D,E represent bivariate relationships between the fundamental variables. Figure 2D of the scatter plot of the development of sports services and the development of the silver economy indicates that there is an evident positive correlation with the increase in the values of the sports services index with the increase in the levels of silver economy development. Even though dispersion rises as the index values become larger, the overall increasing tendency confirms the hypothesis according to which sports services can be viewed as enabling factor of the aging-related economic activity. The results of Figure 2E also show that there is a close positive correlation between the development of sports services and the physical participation of older adults in physical activities. The participation rates are also significantly higher in areas with better sports service systems, which highlights the behavioral channel whereby the sports services can have a downstream effect to the economy. These observed relationships are further strengthened by the correlation analysis. Table 3 demonstrates that both the silver economy development index and physical activity participation of older adults has a positive relationship with the index of sports services development. Sports services and physical activity are more correlated, as it appears in line with theoretical prediction in such a way, that service supply impacts exercise behavior directly, and economies outcomes are more results of cumulation. The following application of the dynamic interaction models explicitly based on the consideration of the behavioral mechanisms and the bidirectional causality can be justified by these patterns of correlations. The descriptive and correlation findings, in general, develop three critical empirical assumptions in the forthcoming analysis. All of these such as sport services development, participation in physical activity among senior citizens and growth of the silver economy will be systematically related and not independently distributed. Demographic aging is linked to significant structural variations of participation behavior. The associations observed are sufficiently strong and consistent enough to justify the formulation of the interactive development paths by some form of dynamic modeling.

**Table 3:** Pearson correlation coefficients among the main indices.

	Sports services index	Silver economy index	Physical activity rate
Sports services index	1	0.32	0.61
Silver economy index	0.32	1	0.14
Physical activity rate	0.61	0.14	1

### 3.2 Construct reliability and validity of the measurement model

The reliability and validity of the measurement model were evaluated prior to estimating the dynamic interaction models to ensure that the latent constructs of sports services development and the silver economy were empirically sound (25). Confirmatory factor analysis was conducted using the predefined indicator structure, and multiple complementary criteria were applied to assess internal consistency, convergent validity, and discriminant validity.

**Figure 3:**
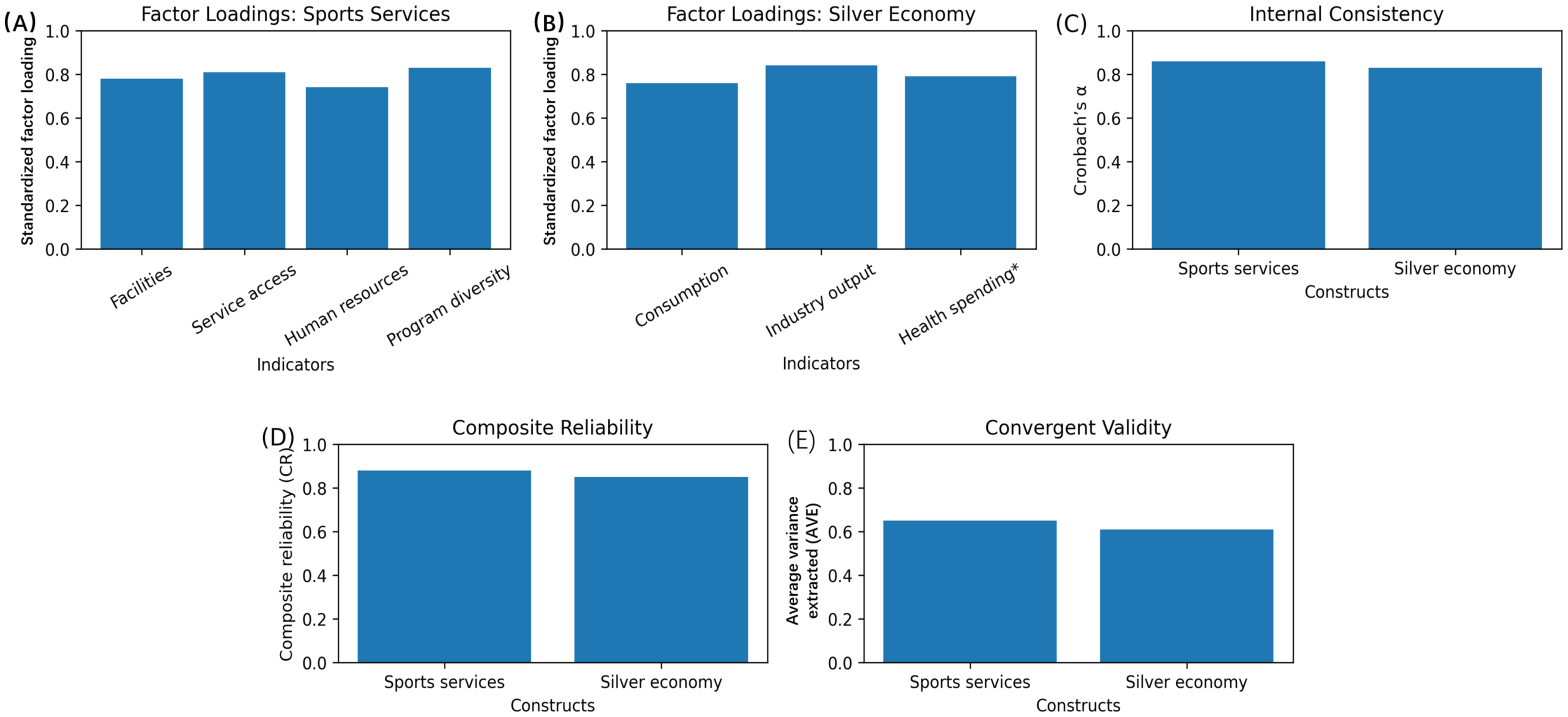
Measurement model and construct validation. **(A)** Standardized factor loadings for the sports services development construct (facilities, service accessibility, human resources, and program diversity). **(B)** Factor loadings for the silver economy construct (older adults’ consumption, industry output, and health-related expenditure). Cronbach’s *α* **(C)** and composite reliability **(D)**, respectively. **(E)** Average variance extracted (AVE).

In Figure 3A, the standardized factor loadings of construct of sports services development were given, and it was modeled as a multidimensional latent variable including facility availability, service accessibility, human resource and diversity of programs. Factor loading is greater than the standard-accepted number of 0.70, suggesting that every perceived indicator makes a significant contribution to the concepts behind it. Human resources is the most loaded, indicating the significance of professional staffing in realizing the functionality of sports services, and that the density of facilities and accessibility of services have significant and balanced contributions (26). The standardized factor loadings for the silver economy construct based on older adults’ consumption shown in Figure 3B, aging-related industry output, and a health-spending indicator defined to reflect wellness/preventive and health-maintenance demand. The comparatively strong loading of this component indicates that health-maintenance-oriented spending is a central dimension of the silver economy in our measurement model, which aligns with the conceptualization of sports services as a form of preventive health infrastructure. Loadings are moderate and high and prove the idea of the silver economy as a complex economic system instead of a one-dimensional result. Internal consistency reliability is presented in Figure 3C. The relatively high loading that is related to health-related expenditure demonstrates the way the consumption of products is concentrated to the values of health-related expenditure in the economic system of aging civilizations. The Cronbach’s 0.80 values of the two constructs are above satisfactory, which indicates good internal consistency, and it shows that the chosen indicators are good at measuring their respective latent variables. Figure 3D on composite reliability values also validates this evaluation. The values of all values exceed the recommended value of 0.70, which gives the further assurance that the constructs are not being overwhelmed by measurement error. The average variance extracted (AVE) was used to determine convergent validity as indicated in Figure 3E. Both the AVE values of the development of the sports services and the silver economy are above 0.50 and that means that both the constructs explain above half the variance in its indicators. This outcome facilitates the suitability of the measurement model in reflecting the targeted theoretical constructs. The Fornell–Larcker criteria was used to test discriminant validity, and it was presented in Table 4. The inter-construct correlations are less than the square roots of AVE, which are shown in the diagonal of the matrix.

**Table 4:** The Fornell–Larcker discriminant validity matrix, with square roots of AVE on the diagonal.

	Sports services	Silver economy
Sports services	0.81	0.52
Silver economy	0.52	0.78

The tendency means that the development of sports services and the silver economy are empirically differentiated constructs even though they are conceptually and empirically related. The finding alleviates the risk of redundant measures of constructs and encourages the utilization of distinct latent variables in further interaction and dynamic examination. Taken together, the measurement model shows high reliability and validity in a variety of parameters. Both the multidimensional structure of the sports services, as well as that of the silver economy are empirically validated to give the results of the study a strong foundation in estimating dynamic, bidirectional relationships in the results sections that follow.

### 3.3 Directional effects and mediating mechanisms between sports services and the silver economy

**Figure 4:**
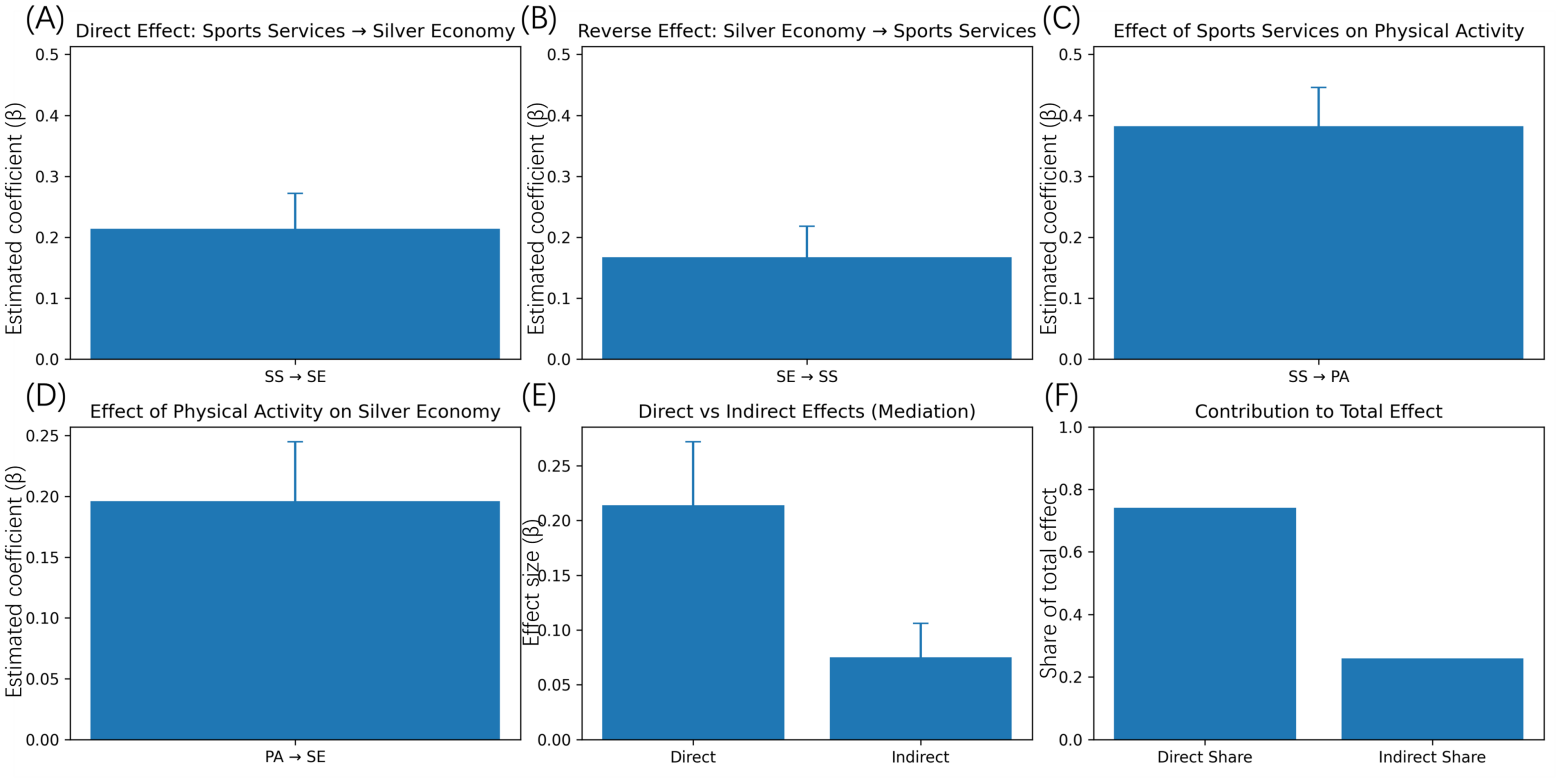
Main directional effects and mediation results. **(A)** The direct effect of sports services development on silver economy growth. **(B)** The reverse effect of silver economy development on sports services. **(C)** The effect of sports services development on older adults’ physical activity participation. **(D)** The effect of physical activity participation on silver economy development. **(E)** The total effect of sports services on the silver economy into direct and indirect components. **(F)** The relative contribution of direct and indirect effects. Error bars represent standard errors.

The main dynamic effects estimated from the reciprocal panel models provide clear evidence of directional linkages between sports services development and the silver economy. Figure 4A reports the estimated direct effect of sports services development on silver economy growth. The coefficient is positive and statistically significant, indicating that regions with higher levels of sports service provision in the previous period tend to experience stronger subsequent expansion of aging-related economic activities. In substantive terms, improvements in sports services are associated with increased consumption and industry output linked to health, leisure, and wellness, suggesting that sports services function as a productive input into the silver economy rather than merely a public expenditure item (27). Figure 4B presents the reverse pathway, examining whether silver economy development feeds back into the sports services system. The estimated coefficient is also positive and statistically significant, though slightly smaller in magnitude than the forward effect. This result indicates that market expansion and increased spending by older adults generate demand-side pressure for the expansion and upgrading of sports services. Regions with more developed silver economies appear more capable of supporting diversified programs, professional staffing, and improved service accessibility, consistent with a demand-driven service evolution mechanism.

To explain the behavioral pathway of the two systems, the impact of sports services on the participation in physical activity among older adults was approximated individually. Figure 4C indicates that sports services development has a firm positive impact on the practice of physical activities among older adults. The scale of this effect is larger than is experienced in the direct economic pathway, which serves to highlight the importance of service provision in knowledge and practice in the designed exercise behavior. This observation aligns with the fact that available facilities, expert consultation and the diversity of the programmes reduce the barriers to participation, and facilitate continued interest in physical activity (28). The development of silver economy emotional event of physical activity participation is estimated to give Figure 4D. The coefficient is positive and statistically significant, which means that an increase in the extent of physical activity of older adults would result in further growth in the economic activity of the aging process. It is also probable that this association indicates the direct consumption impacts like expenditure on fitness and wellness services as well as the indirect impacts concerning enhanced functional health and expanded involvement in social and personal activities. Figure 4E represents the summarized mediation of the physical activity participation. Individual breakdown of the overall impact of sport services on the silver economy demonstrates that along with significant direct impact, the significant part of impact is working through the enhancement of participation in physical activities. Indirect effect was calculated doing a bootstrap test with region resampling to get the bootstrap and the confidence interval does not include zero which statistically supports the mediation pathway. Figure 4F shows the proportion between the direct and indirect channels of the total effect. Although it is the direct pathway which explains most of the impact, there is an indirect pathway that is not a negligible proportion through the participation in physical activities. This trend suggests that sports services provide a contribution to the development of the silver economy by means of directly stimulating aging-related market and improving the health-related behaviors that can support the higher levels of economic activity.

Supplemented by these results, it is possible to prove that there is a bidirectional, behaviorally mediated relationship between sports services and silver economy. The sports services do not only adapt to the expansive aging market, but it also directly impacts it by influencing participation in sports activities and consumption behaviors with regards to health. The results are empirical evidence of the fact that sports services and the silver economy should be modeled as interacting systems and not domains by themselves.

### 3.4 Interactive and dynamic development effects

**Figure 5:**
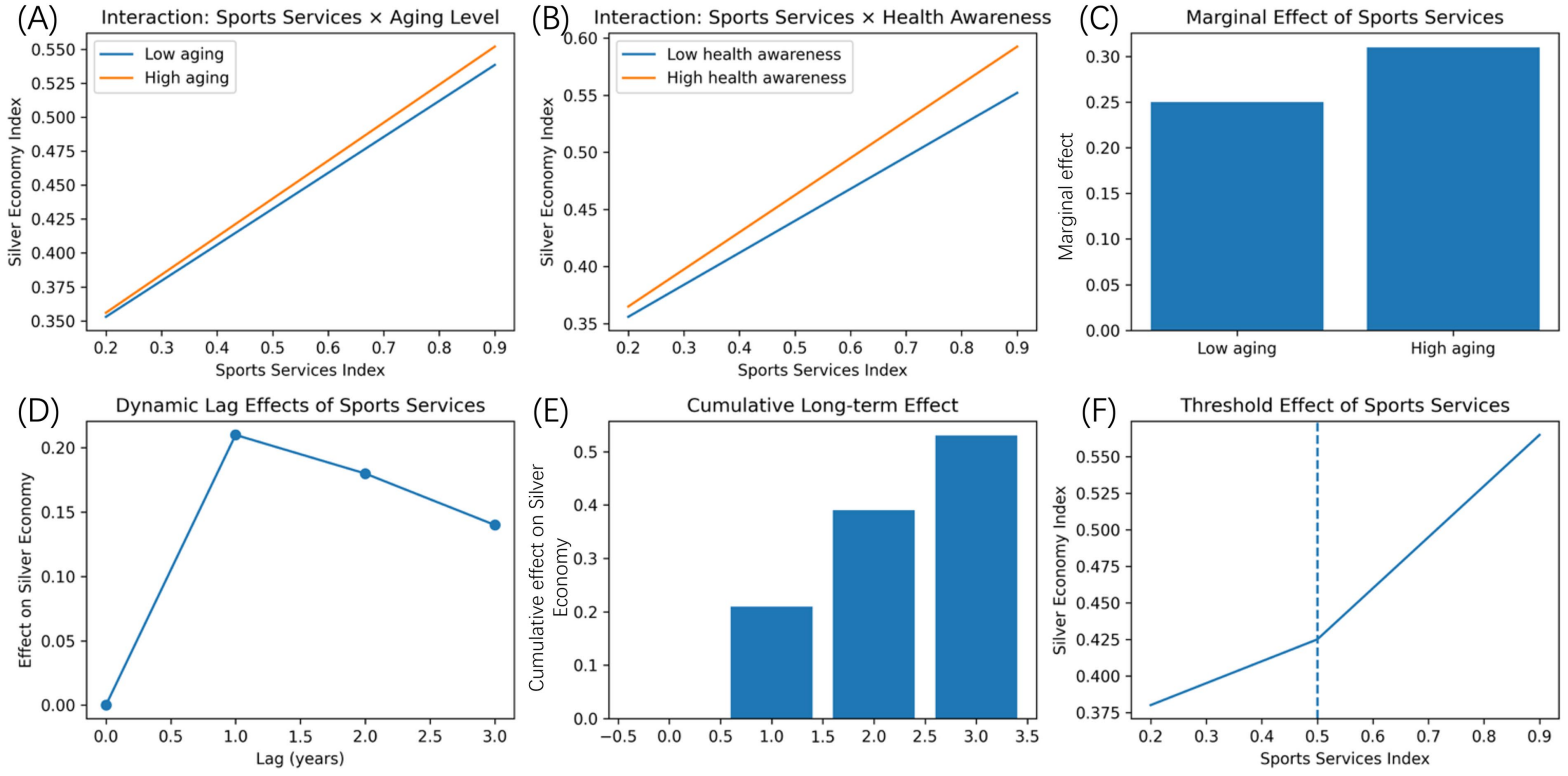
Interactive and dynamic development effects. **(A)** The interaction between sports services development and population aging level. **(B)** The interaction between sports services development and health awareness. **(C)** Marginal effects under different aging conditions. **(D)** Lagged effects of sports services development on silver economy outcomes. **(E)** Cumulative effects across lags. **(F)** A threshold relationship between sports services development and silver economy growth.

The interactive and dynamic specification gives an additional understanding of how the impact of the sports services development on the silver economy depends on the demographic and behavioral situation and changes over time. The summarized terms of the estimated interaction, lag structure, and non-linear patterns obtained after the dynamic panel models are represented in Figure 5. Figures 5A,B reveal the effects of interaction between the sports services development and the main contextual variables. As revealed in Figure 4A, the degree to which the sports services have the marginal effect on the development of the silver economy varies systematically depending on the level of population aging. The relationship between sports services and the development of the silver economy has an increasing steep slope in an area where the number of older people is higher, and thus, the better the sports services are, the greater the economic benefits of the situation will be in the city with more older people. Such a trend indicates that, the productivity of sports services is determined by demographic structure where the intensity of aging increases the demand-side returns and health-related returns.

The same moderating tendency can be seen in health awareness which is shown in Figure 5B. Those areas with greater density of health awareness have a greater correlation of sports services development and outcomes of silver economy. This finding implies that sports services are better in creating economic spillovers in cases where the older adults are more receptive to the health-related information and more willing to convert service availability into active use and associated consumption. The heterogeneity of these terms of interaction is summarized in Figure 5C which records the marginal effects of sports services that are estimated given various demographic conditions. This shows that the effect size is significantly greater in high-aging areas than in low-aging areas, which would have confirmed the assumption of uniform effects would hide significant structural differences across populations. The dynamic effect is reported in Figures 5D,E. Figure 5D is the lagged effect of the development of sports services on the silver economy in a number of periods. Its impact is highest at the first lag and decreases rapidly in the later periods (29), which means that the improvements in sports services can have the most effective effect in the short- and medium-term, but still, the effect is statistically significant in the long-term. This time trend is in line with time period needed to have service upgrades to influence participation levels, consumption habits and industry reactions. Figure 5E records the development in one lags cumulative sports services. Although the marginal effect decreases as time goes, the same effect is accumulating and proves evidence of a path dependence and health capital accumulation. This observation implies that long-term economic gains in sports services are long-term and not short-term. Figure 5F investigates possible non-linearities by modelling a threshold relationship between the development of sports services and the results of the silver economy. Under a specific level of development, the benefits are less economic growth related to improvements in sports services. At a point where the index of sports services crosses the identified threshold, the slope of the association rises significantly. This trend suggests that there exists some coordination or scale threshold, above which sport services are enough comprehensive to achieve sustained involvement, behavioral change, and economic fashion.

Combined, these findings confirm that the correlation between sports services and the silver economy is not linear and constant. It is dynamically changing in nature and non-linear as the extent of the effect varies based on the nature of the demographic setup and the level of health consciousness. These characteristics indicate that interaction effects and time dynamics need to be considered when evaluating the role of sports services in the aging societies.

### 3.5 Heterogeneity in regional contexts and service structures

**Figure 6:**
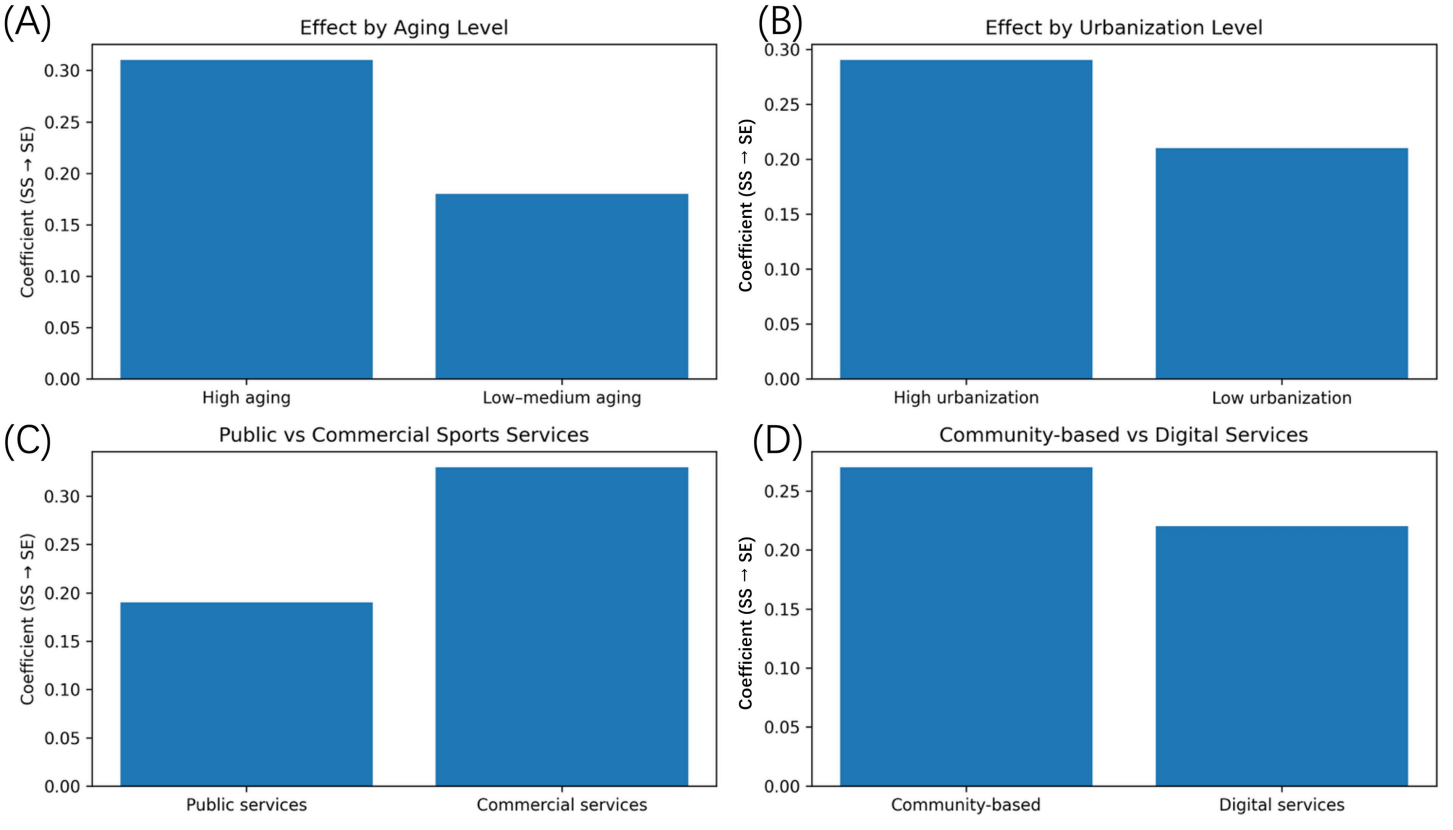
Heterogeneity analysis of sports services effects. **(A)** Estimated effects of sports services development on silver economy outcomes in regions with high versus low-to-moderate aging levels. **(B)** Compares regions with high and low urbanization levels. **(C)** Contrasts public and commercial sports services. **(D)** Compares community-based and digital sports service modes. Coefficients are derived from subgroup-specific dynamic panel models.

In order to further investigate the hypothesis of whether the impacts of services development in the sport sector vary by the contexts of the regions and service structures, the heterogeneity of the sample was done by stratifying the sample based on important demographic, spatial, and institutional variables. Figure 6 summarizes the estimates of the coefficients by subgroup-specific models of the effect of sports services development on the results of the silver economy. Figure 6A provides the comparison between the regions where aging intensity is high and the ones with low-to-moderate levels of aging. The estimated coefficient of the sports services development is significantly higher in areas of high age showing that the economic returns to sports services are more intense in the regions where older adults are more prevalent in the population composition. This finding indicates that the effect of demographic pressure increases the demand of both health oriented and leisure related goods and services, and that enhancements in the sports services would have a greater likelihood of cutting into growth in the silver economic growth. Figure 6B shows heterogeneity in terms of urbanization. The impacts of the development of sports services in the silver economy are stronger in the areas that are highly urbanized than in the lesser urbanized areas. Such trend probably indicates variations in the infrastructure concentration, accessibility of services and market integration. In the environments that are highly urbanized, the services of sports might be complementary to the transportation systems, business premises, and network of information technologies, which contributes to the increased ability to promote the aging-related consumption and growth in the industry.

The heterogeneity of service structure occurs in Figure 6C in the separation between the public and commercial sports services. Commercial sports services have a larger effect on the case of the other estimations than the service public. Although the importance of public sports services in offering access to the baselines and equity cannot be underestimated, the commercial services seem to be more adequate to the market demand and can produce the direct economic spillover. This observation brings out the distinction of service providers in the overall sports services framework. Figure 6D seeks to compare community-based sports services with digital and technological-assisted forms of services. The community based services show a closer relation with development of a silver economy which implies that the proximity, social interaction and continuity of the service is especially critical to the older adults. Although digital sports services are also positively related to the economic outcomes, the extent of their impact on the latter is smaller, which suggests that technological solutions could serve as an addition instead of a substitute of community-based service delivery in aging communities.

Overall, the heterogeneity analysis demonstrates that the impact of sports services development is context-dependent. Demographic structure, urban environment, and service organization shape both the magnitude and channels through which sports services contribute to silver economy growth. These findings reinforce the importance of moving beyond average effects and accounting for structural differences when evaluating the role of sports services in aging societies.

### 3.6 Model fit, robustness, and sensitivity checks

**Figure 7:**
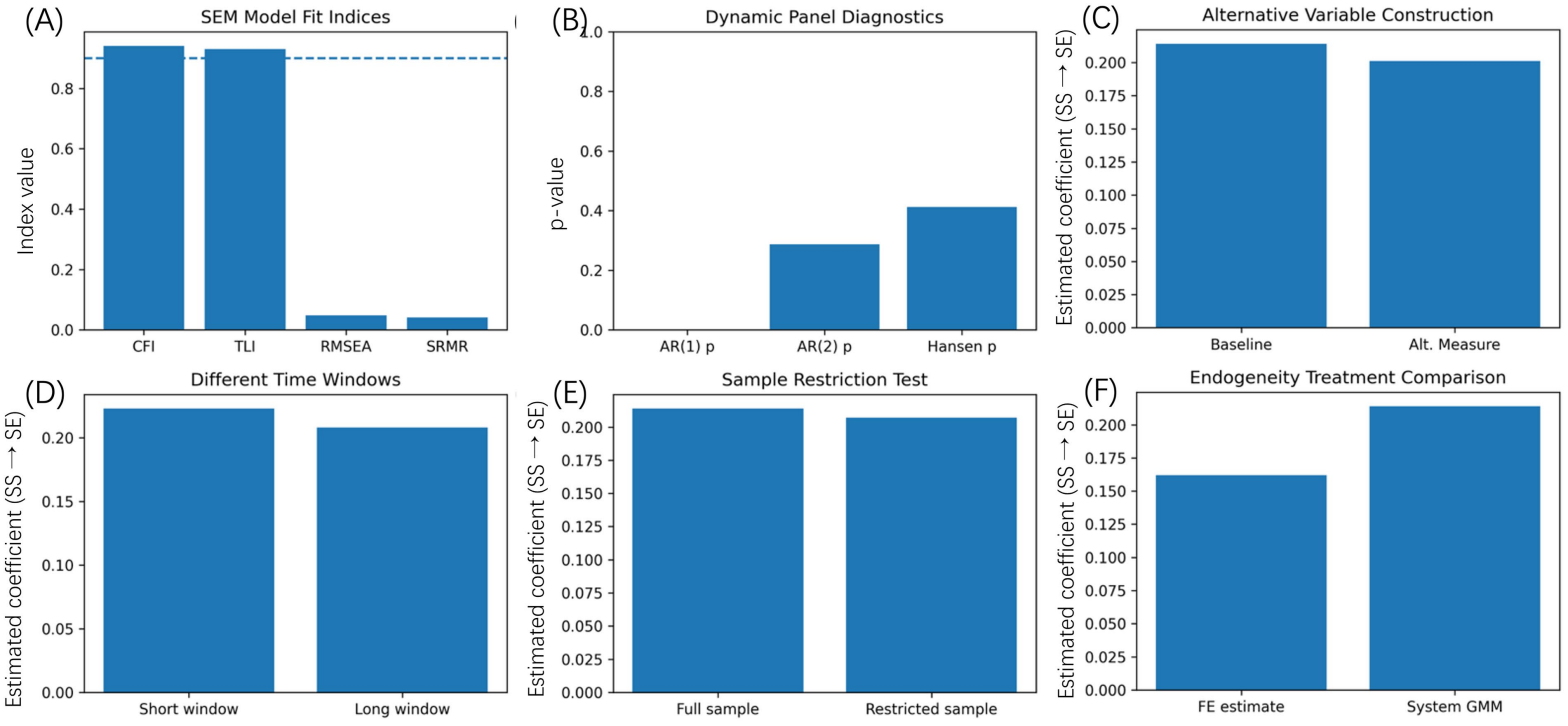
Model fit, robustness, and sensitivity checks. **(A)** Structural equation model fit indices (CFI, TLI, RMSEA, SRMR). **(B)** Piagnostic statistics for dynamic panel estimation (AR(1), AR(2), Hansen test). Robustness checks using alternative variable construction **(C)**, different time windows **(D)**, and restricted samples **(E)**. **(F)** Compares baseline fixed-effects estimates with system GMM estimates addressing endogeneity.

Adequacy of models and stability of results were tested in a sequence of complementary diagnostic, robustness and sensitivity analysis. The checks were carried out to determine whether the estimated relationships are based on the model misspecification, sample composition, or endogeneity instead of the empirical parameters remaining constant. Fit index of the structural equation model of validation of the measurement structure and directional paths were reported in Figure 7A. The both the comparative fit index (CFI) and Tucker–Lewis index (TLI) have values that exceed the generally accepted values, and this shows a good overall model fit. Root mean square error of approximation (RMSEA) and standardized root mean square residual (SRMR) are substantially lower than standard cut-off values indicating that the given model can replicate the observed covariance structure with a minimal approximation error. A combination of all of these indicators shows that the structural relationships between latent constructs are sufficiently described.

Figure 7B shows the diagnostic statistics of the dynamic panel estimations. The Arellano–Bond test means the first-order that is anticipated to be correlated with the second-order serial correlation is considered to be non-evidential. The Hansen test of over-identifying restrictions has a *p*-value range that is acceptable thereby indicating the joint validity of the set of instruments. It indicates that these diagnostics determine that the dynamic specifications have been identified in an appropriate way, and instrument proliferation has not internally distorted the estimates. Strength against construction of other variables is demonstrated in Figure 7C. In a case whereby the core sports services and the silver economy indices are rebuilt with other available aggregation processes, the approximate impact of sport services development on the outcome of a silver economy is positive and within similar magnitude to the initial estimate. This consistency implies that the key findings have no particular index construction strategy. Figure 7D looks at sensitivity to various time windows. The re-estimation of the models with shorter and longer sample periods gives a comparable coefficient to the baseline findings. Even though there are slight differences in severity, the direction, and statistical significance of the main relationships remain consistent indicating that the outcome of the results are not echoed by the arbitraryness of the observation window.

Further strength is evaluated by sample restriction tests which are demonstrated in Figure 7E. The omission of areas with extreme values of economic or demographic variables also has no substantial effect on the estimated effects. The fact that this finding is achieved suggests that the outcomes are not influenced by a limited number of influential observations. Lastly, Figure 7F compares fixed effects baseline estimates with system GMM estimates that explicitly deal with endogeneity due to reverse causality and dynamic persistence. Although the fixed-effects model estimates the coefficient coefficients smaller than from system GMM (30), it gives a consistent direction and the same relative magnitude. The greater estimates with system GMM are also replicable with attenuation bias with respect to the presence of lagged dependent variables which support the suitability of the dynamic panel methodology.

All these factors make the findings of the empirical study reliable, overall, but strong support can be made in regard to model fit analysis, robustness test, and endogeneity treatment. The relationships between the development of sports services, the physical activity and growth of silver economy observed are strong in alternative specification and estimation strategies.

### 3.7 Dynamic responses of the silver economy: impulse response analysis

**Figure 8:**
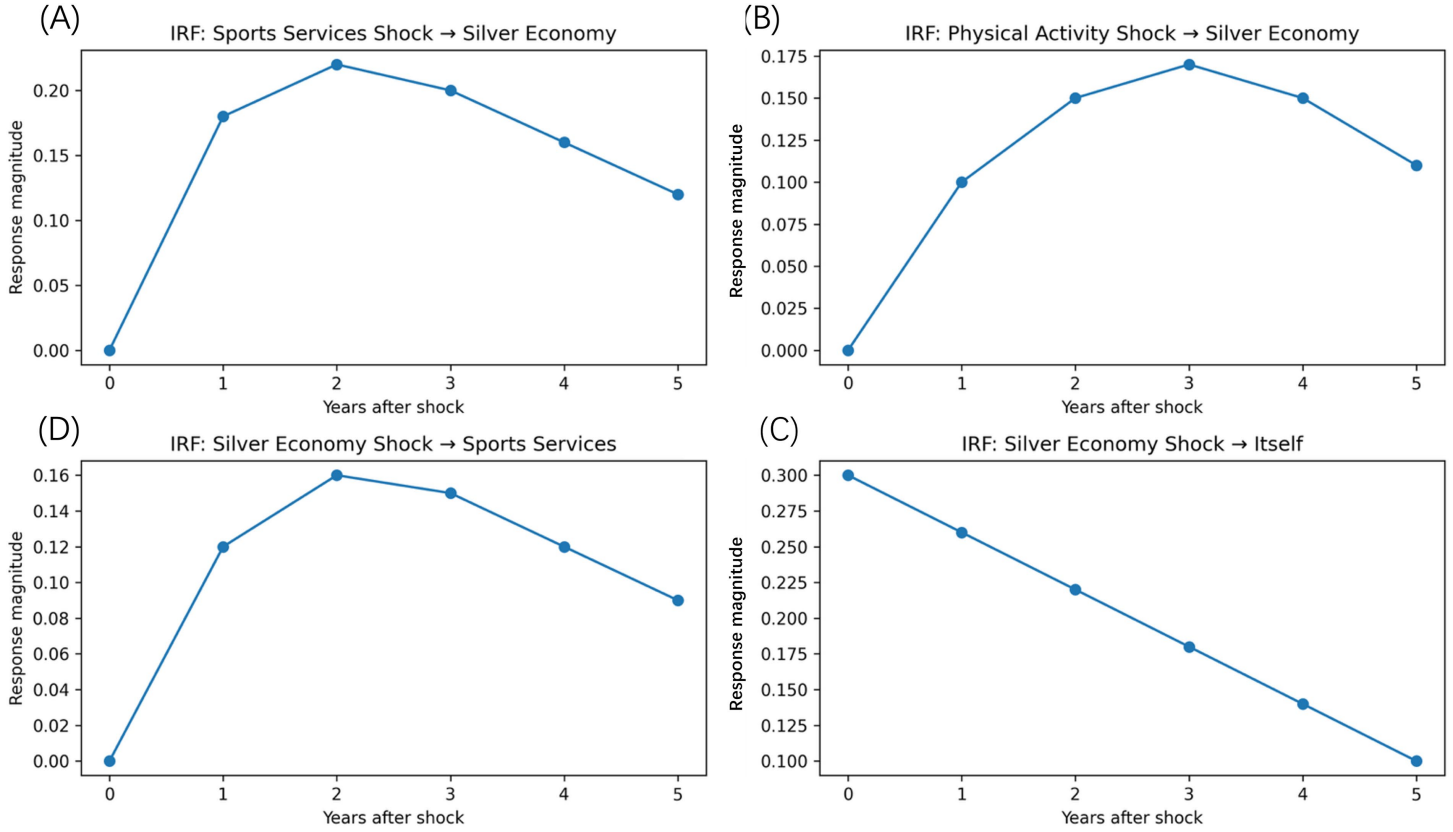
Impulse response functions related to the silver economy. **(A)** The response of the silver economy to a positive shock in sports services development. **(B)** The response to a shock in older adults’ physical activity participation. **(C)** The response of sports services development to a silver economy shock. **(D)** The persistence of the silver economy’s own shock. Impulse responses are derived from the dynamic panel system and plotted over a multi-period horizon.

In order to further understand the dynamic reaction patterns of the estimated relationships, impulse response functions were obtained, based on the dynamic system to trace the temporal impacts of exogenous shocks in the silver economy. Figure 8 shows the time-dependent response of the silver economy to the shocks in the development of sports services, the number of people who participate in the physical activity and to its dynamics.

In Figure 8A, the silver economy is created to respond to a unitary positive shock on the development of sports services. The reaction is not acute, but after one period, it is acute and peaking in the short-to medium term, then it is decaying. This trend demonstrates that information regarding sports services takes time to have contributions in terms of participation behavior, consumption pattern and industry activity, but once this happens, the economic effects are lasting in the course of multiple phases. Its slow fading away over time indicates that the effects are not immediate in nature, but instead cumulative, as the process of the health capital accumulation process would imply. Figure 8B represents the impulse reaction of the silver economy to a shock on the participation of older adults in physical activity. The reaction is of a rather inertial and lagging nature, which supports the conclusion of the behavioral changes as a channel of transmission between sport services and the economic consequences. It also seems that increased physical activity is an area that contributes to silver economy development by promoting functional health, prolonging engagement in leisure and wellness activities, and maintaining the consumption ability of older people.

Figure 8C shows the silver economy feedback pathway on the sports services. An increase in the price of silver has a significantly lower effect on the development of sports services, but a more pronounced effect results in an increase and a faster decline in the effect compared to the forward effect. Such an asymmetry implies that as the market is enlarged and consumption grows, the service upgrading can be triggered, whereas the supply-side reaction is restrained more by the institutional and infrastructural adjustment costs. Figure 8D records the reaction of the silver economy to a shock to the economy and this indicates the extent to which the system is persistent and path dependent. The slowly fading reaction suggests that the development of silver economy is characterized by a high inertia and the existing growth tendencies are formed in part due to the previous directions. Such a persistence contributes to understanding that short-term interventions can possibly have little immediate effects and that long-term development paths can be changed only with the assistance of long-term coordinated investment. Generally, the impulse response analysis is a good complement to the regression-based results since it exposes the temporal pattern of the interaction of the interaction of sports services and the silver economy. These findings highlight once again that the association is dynamic, asymmetric, and has delayed yet long-lasting impact, and support the idea of sports services as a sustainably increasing (over time) driver of age-related economic growth, as opposed to a temporary booster.

## 4 Discussion

### 4.1 Integrating the findings into a coherent mechanism

A central contribution of this study is to treat sports services not only as recreational provision but as a component of preventive health infrastructure that can support health-capital formation over time. This interpretation is consistent with ecological models of physical activity, which emphasize that built and organizational environments shape the opportunity structure for sustained participation, and with health capital and life-course perspectives that view functional capacity as an accumulative stock influenced by repeated behavioral investments (31). Under this framing, sports services affect downstream aging-related economic activity primarily by changing the conditions under which older adults can maintain regular physical activity and preserve functional capacity. The empirical patterns are therefore interpreted as consistent with a mechanism in which service provision supports long-term capability maintenance, which then underpins participation in health- and leisure-related consumption and activities associated with the silver economy.

The bidirectional logic is also theoretically plausible beyond the empirical modeling strategy. Sports services can increase demand for aging-related markets through improved participation capacity and health-maintenance preferences, while silver-economy expansion can generate derived demand and resource capacity that feed back into service upgrading (32). This feedback perspective helps move beyond a one-directional “services → outcomes” narrative by recognizing that market growth and service systems may co-evolve through mutually reinforcing incentives. In this sense, bidirectionality reflects a combined supply-demand adjustment process, where the pace and strength of each pathway depend on institutional capacity, service organization, and the responsiveness of providers.

### 4.2 Behavioral pathways and the role of physical activity as an observable mediator

The mediation framework highlights that infrastructure and service inputs do not translate into economic outcomes automatically; behavioral responses are the proximate channel through which service systems become economically consequential. Ecological and behavioral models of sports and exercise participation suggest that accessibility, social support, and perceived competence are key determinants of sustained engagement, all of which can be influenced by how sports services are designed and delivered (33). This perspective is useful for interpreting why service expansion may generate limited downstream effects when programs are poorly matched to older adults’ needs or when participation barriers remain high despite infrastructure growth.

At the same time, physical activity participation should not be interpreted as the sole mechanism. It is more defensible to view physical activity as an observable indicator within a broader constellation of lifestyle and social participation processes linked to sports services. Participation in organized sports services can co-occur with improvements in health literacy, preventive self-management routines, and community social connectedness, which may also shape aging-related spending and service demand. Because the panel data capture physical activity more directly than these adjacent mechanisms, the mediation evidence should be interpreted as conservative support for a behavior-linked pathway rather than a complete decomposition of all underlying channels.

### 4.3 Context dependence, threshold features, and service-system complementarity

The heterogeneity and interaction patterns are consistent with the idea that the productivity of sports services is context dependent. In settings with stronger demographic aging pressure, the demand for health-maintenance activities is structurally higher, increasing the likelihood that improvements in sports services translate into sustained engagement and related consumption. Similarly, higher health awareness can amplify behavioral responsiveness to service availability, reinforcing the view that sports services operate as a complementary input whose returns depend on broader social and demographic conditions.

Nonlinear and threshold-like patterns provide an additional interpretive layer. From an ecological standpoint, fragmented or low-intensity service provision may fall below a minimum effective scale required to generate meaningful participation change. Once service systems reach a level of coverage, professionalism, and program continuity that reduces participation frictions, downstream effects can become more pronounced. This helps explain why investments in facilities alone sometimes fail to produce expected health or economic benefits and underscores the importance of service quality, program design, and user engagement rather than infrastructure expansion per se.

### 4.4 External validity and institutional considerations

The evidence in this study is derived from regional variation within a single national context, which implies a shared institutional framework shaping both sports-service provision and aging-related markets. While the proposed mechanisms are relevant to aging societies more broadly, effect magnitudes—and potentially the relative strength of feedback pathways—may differ under alternative institutional arrangements (34). Cross-national generalization should therefore emphasize mechanism plausibility rather than assuming transferable parameter estimates.

Institutional differences may alter the observed dynamics in predictable ways. Under more generous welfare regimes with strong public provision of preventive health services, the marginal contribution of sports services to aging-related markets may be smaller because baseline access and health support are already high, although such settings may strengthen equity effects and reduce threshold barriers. In more market-oriented systems, commercial providers and private insurers may respond more rapidly to aging-related demand, potentially intensifying the feedback loop from silver-economy expansion to service upgrading while also increasing access disparities. Sports governance structures may further shape dynamic responsiveness: centralized governance can facilitate coordinated scaling and standardization, whereas decentralized or community-led governance may strengthen participation-based mechanisms through local social capital but produce more uneven regional development. These institutional contrasts indicate that future comparative work is necessary to test whether similar dynamics emerge across different governance and welfare configurations.

### 4.5 Limitations and future research

Several limitations should be considered when interpreting the findings. First, the empirical analysis relies on observational panel data; although lag structures, fixed effects, and system GMM reduce simultaneity and endogeneity concerns, time-varying unobserved factors cannot be fully ruled out, and results should be interpreted as conditional associations consistent with the proposed framework rather than definitive causal effects. Second, the physical activity mediator is measured using a harmonized “regular exercise” criterion, but it may still imperfectly represent the broader behavioral and social participation mechanisms that link sports services to aging-related economic activity. Third, the study is conducted within a single national context; institutional features may shape both baseline service provision and market responsiveness, limiting direct cross-country generalization. Future studies could strengthen causal inference using quasi-experimental designs, incorporate richer behavioral batteries (e.g., health literacy, preventive care engagement, social connectedness), and test institutional moderation through cross-national comparative datasets.

Because the analysis uses region-level observational panel data, the estimated effects should be interpreted as associations at the aggregate level that are consistent with the proposed causal framework rather than definitive causal effects. In particular, ecological inference is subject to the risk of ecological fallacy, whereby relationships observed between regions may not map directly onto individual-level behavioral or economic processes. Although lag structures, fixed effects, and system GMM help mitigate simultaneity and some forms of endogeneity, unobserved time-varying confounding and aggregation bias cannot be fully excluded. Future research combining individual-level longitudinal data with regional service indicators (multi-level designs) or quasi-experimental variation would strengthen causal interpretation and reduce ecological inference concerns.

## 5 Conclusion

Sports services appear to have a structurally relevant role in aging societies by linking health promotion to the expansion of aging-related markets. Rather than being limited to recreational provision, sports services can be viewed as part of a broader service system that is associated with older adults’ physical activity participation and with economic activity in silver-economy domains. Using regional panel evidence from China, this study documents dynamic and bidirectional associations between sports services development and silver economy development, and highlights physical activity participation as an empirically observable behavioral pathway consistent with a preventive-health interpretation.

More broadly, the study contributes to research on aging, physical activity, and service systems by showing that the relationships between sports services and silver-economy outcomes are context-dependent, behavior-linked, and persistent over time. These results support continued investigation of sports services as a potential component of preventive health systems with economic relevance, while underscoring the need for future work using richer behavioral measures and cross-national designs to test whether similar patterns and effect magnitudes hold under different welfare regimes and sports governance structures.

## Data Availability

The original contributions presented in the study are included in the article/Supplementary material, further inquiries can be directed to the corresponding author.
