# Supplementary material for: Modeling the interactive development of sports services and the silver economy in aging societies
Source: Front Public Health. 2026 Mar 31;14:1777277. doi: 10.3389/fpubh.2026.1777277 (PMC13076113; doi:10.3389/fpubh.2026.1777277)
Supplement: Supplementary file 1 [file Table_1.DOCX]

**Supplementary information**

**For**

**Modeling the Interactive Development of Sports Services and the Silver Economy in Aging Societies**

**Table S1. System GMM diagnostics and instrument count.**

| **Model** | **Dependent variable** | **Key regressor** | **Coefficient** | **Std. Error** | **p-value (coef)** | **AR(1) p-value** | **AR(2) p-value** | **Hansen J p-value** | **Number of instruments** | **Number of groups (regions)** | **Observations** | **Instrument strategy** |
| --- | --- | --- | --- | --- | --- | --- | --- | --- | --- | --- | --- | --- |
| Eq.(2) Main effect | Silver Economy Index | Sports Services Index (t-1) | 0.214 | 0.058 | 0.0003 | 0.001 | 0.287 | 0.412 | 18 | 31 | 372 | System GMM; collapsed instruments; lag(2..3) for endogenous vars |
| Eq.(1) Reverse effect | Sports Services Index | Silver Economy Index (t-1) | 0.167 | 0.051 | 0.0012 | 0.002 | 0.301 | 0.455 | 18 | 31 | 372 | System GMM; collapsed instruments; lag(2..3) for endogenous vars |
| Mediation step | Physical Activity Rate (60+ %) | Sports Services Index (t-1) | 0.382 | 0.064 | 0 | 0.001 | 0.276 | 0.398 | 17 | 31 | 372 | System GMM; collapsed instruments; lag(2..3) for endogenous vars |
